# The genome sequence of a cranefly *Tipula* ( *Lunatipula*) *vernalis *Meigen, 1804

**DOI:** 10.12688/wellcomeopenres.23203.1

**Published:** 2024-11-07

**Authors:** Olga Sivell, Duncan Sivell, Wellcome Sanger Institute Tree of Life Management, Samples and Laboratory team, Wellcome Sanger Institute Scientific Operations: Sequencing Operations, Wellcome Sanger Institute Tree of Life Core Informatics team, Tree of Life Core Informatics collective, Darwin Tree of Life Consortium

**Affiliations:** 1 Natural History Museum, London, England, UK

**Keywords:** Tipula vernalis, cranefly, genome sequence, chromosomal, Diptera

## Abstract

We present a genome assembly from an individual female cranefly *Tipula vernalis* (Arthropoda; Insecta; Diptera; Tipulidae). The genome sequence has a total length of 1,272.30 megabases. Most of the assembly is scaffolded into 4 chromosomal pseudomolecules. The mitochondrial genome has also been assembled and is 16.01 kilobases in length. Gene annotation of this assembly on Ensembl identified 13,911 protein-coding genes.

## Species taxonomy

Eukaryota; Opisthokonta; Metazoa; Eumetazoa; Bilateria; Protostomia; Ecdysozoa; Panarthropoda; Arthropoda; Mandibulata; Pancrustacea; Hexapoda; Insecta; Dicondylia; Pterygota; Neoptera; Endopterygota; Diptera; Nematocera; Tipulomorpha; Tipuloidea; Tipulidae; Tipulinae; *Tipula*; *Lunatipula*; *Tipula* ( *Lunatipula*) *vernalis* Meigen, 1804 (NCBI:txid2741129).

## Background

*Tipula vernalis* is a western Palaearctic species ranging from northern Portugal and Spain through central and northern Europe to the Urals ( GBIF Secretariat, 2023). In Britian this is a common and widespread cranefly that is often abundant in spring, particularly in May and early June ( Boardman, 2016; Coe, 1950; Stubbs, 1973). *Tipula vernalis* occurs in well-drained grasslands, meadows, road verges and open woodlands and can be common in urban areas, though it tends to avoid very dense vegetation ( Boardman, 2016; Dufour, 1986; Stubbs, 1973; Stubbs, 1992). This cranefly may have an affinity for swards containing sweet vernal-grass, *Anthoxanthum odoratum* ( Stubbs, 2021). Although generally widespread across Britain, there are fewer records of *T. vernalis* in Scotland and this species appears to be scarce north of the Great Glen ( NBN Atlas Partnership, 2024).

*Tipula vernalis* can be recognised in the field by its body colour and wing pattern. The combination of a yellow-brown abdomen with a dark dorsal stripe, orange femora turning black towards the apex, dark brown streaks on the wing, running from the base towards the tip, with smaller white markings nearer the tip, are good indicators of this species ( Boardman, 2016; Stubbs, 1973). The female has a very short ovipositor, an unusual character in British craneflies, and the male has a distinctive v-shaped notch in tergite 9, although genitalia are not required for identification. Both sexes have shining green eyes which, although not unique to *T. vernalis*, is another useful field character, the wing length is 12–17 mm ( Boardman, 2016; Stubbs, 1973; Stubbs, 2021).

Common names proposed for this species include the “green-eyed Tipula” ( Stubbs, 1973) and the “grass long-palp” ( Stubbs, 2021). The larvae and pupae are keyed and illustrated by Brindle (1960) and Podeniene *et al.* (2019).

The high-quality genome of *Tipula vernalis* was sequenced from a single specimen (SAMEA7524261) from Luton, England. The genome was sequenced as part of the Darwin Tree of Life Project, a collaborative effort to sequence all named eukaryotic species in the Atlantic Archipelago of Britain and Ireland.

## Genome sequence report

**Figure 1.**
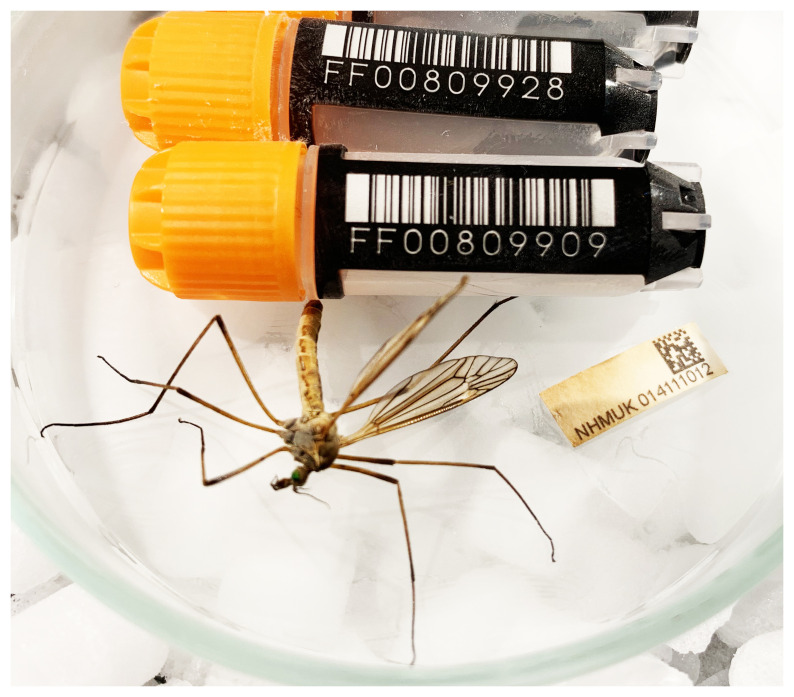
Photograph of the *Tipula vernalis* (idTipVern1) specimen used for genome sequencing.

The genome of an adult *Tipula vernalis* ( Figure 1) was sequenced using Pacific Biosciences single-molecule HiFi long reads, generating a total of 95.93 Gb (gigabases) from 8.76 million reads, providing approximately 44-fold coverage. Primary assembly contigs were scaffolded with chromosome conformation Hi-C data, which produced 142.39 Gb from 942.99 million reads, yielding an approximate coverage of 112-fold. Specimen and sequencing details are summarised in Table 1.

**Table 1.** Specimen and sequencing data for *Tipula vernalis*.

Project information			
**Study title**	*Tipula vernalis*		
**Umbrella BioProject**	PRJEB57430		
**Species**	*Tipula vernalis*		
**BioSample**	SAMEA7524261		
**NCBI taxonomy ID**	2741129		
Specimen information			
**Technology**	**ToLID**	**BioSample accession**	**Organism part**
**PacBio long read sequencing**	idTipVern1	SAMEA7524338	head and thorax
**Hi-C sequencing**	idTipVern2	SAMEA7524346	head and thorax
**RNA sequencing**	idTipVern1	SAMEA7524339	abdomen
Sequencing information			
**Platform**	**Run accession**	**Read count**	**Base count (Gb)**
**Hi-C Illumina NovaSeq 6000**	ERR10489930	9.43e+08	142.39
**PacBio Sequel II**	ERR10499349	2.38e+06	24.08
**PacBio Sequel IIe**	ERR10480610	6.38e+06	71.86
**RNA Illumina NovaSeq 6000**	ERR10489931	6.92e+07	10.46

**Figure 2.**
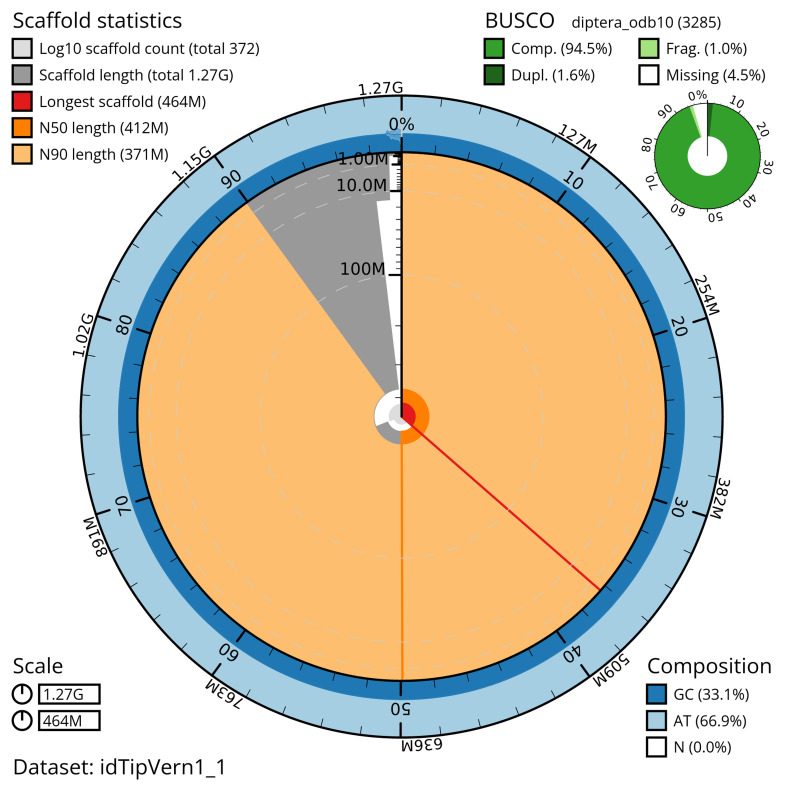
Genome assembly of *Tipula vernalis*, idTipVern1.1: metrics. The BlobToolKit snail plot shows N50 metrics and BUSCO gene completeness. The main plot is divided into 1,000 size-ordered bins around the circumference with each bin representing 0.1% of the 1,272,268,798 bp assembly. The distribution of scaffold lengths is shown in dark grey with the plot radius scaled to the longest scaffold present in the assembly (463,878,994 bp, shown in red). Orange and pale-orange arcs show the N50 and N90 scaffold lengths (411,919,547 and 371,095,977 bp), respectively. The pale grey spiral shows the cumulative scaffold count on a log scale with white scale lines showing successive orders of magnitude. The blue and pale-blue area around the outside of the plot shows the distribution of GC, AT and N percentages in the same bins as the inner plot. A summary of complete, fragmented, duplicated and missing BUSCO genes in the diptera_odb10 set is shown in the top right. An interactive version of this figure is available at https://blobtoolkit.genomehubs.org/view/idTipVern1_1/dataset/idTipVern1_1/snail.

**Figure 3.**
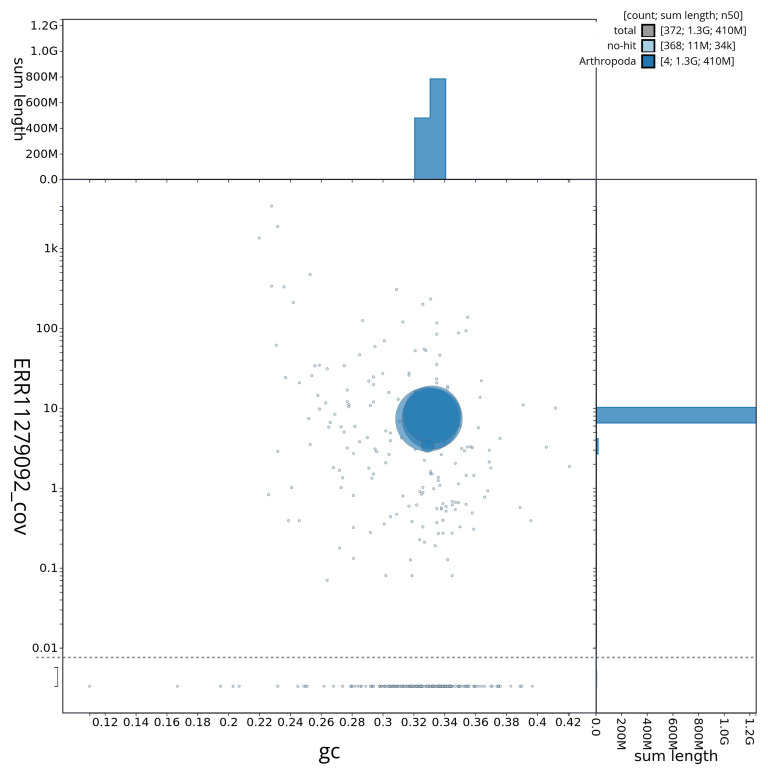
Genome assembly of *Tipula vernalis*, idTipVern1.1: BlobToolKit GC-coverage plot. Sequences are coloured by phylum. Circles are sized in proportion to sequence length. Histograms show the distribution of sequence length sum along each axis. An interactive version of this figure is available at https://blobtoolkit.genomehubs.org/view/idTipVern1_1/dataset/idTipVern1_1/blob.

**Figure 4.**
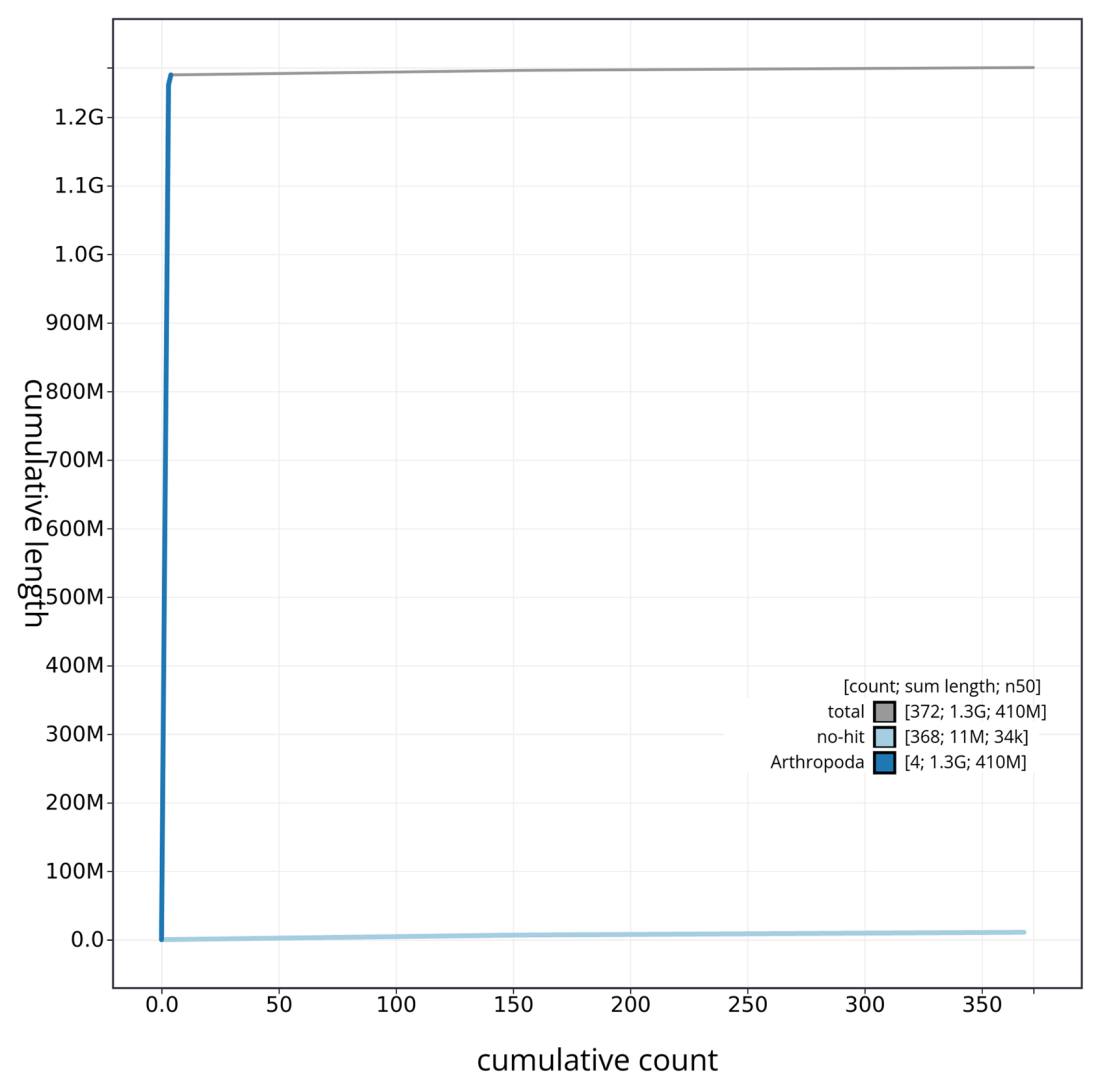
Genome assembly of *Tipula vernalis* idTipVern1.1: BlobToolKit cumulative sequence plot. The grey line shows cumulative length for all sequences. Coloured lines show cumulative lengths of sequences assigned to each phylum using the buscogenes taxrule. An interactive version of this figure is available at https://blobtoolkit.genomehubs.org/view/idTipVern1_1/dataset/idTipVern1_1/cumulative.

**Figure 5.**
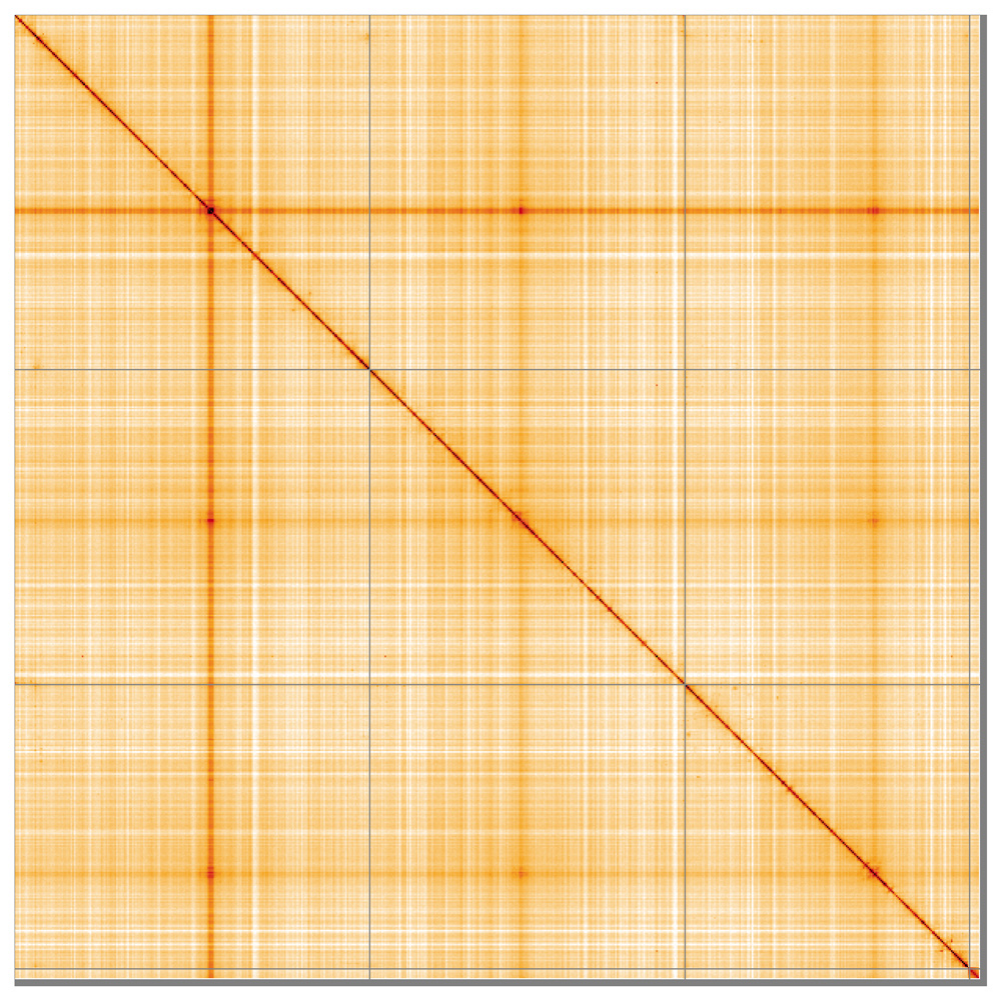
Genome assembly of *Tipula vernalis* idTipVern1.1: Hi-C contact map of the idTipVern1.1 assembly, visualised using HiGlass. Chromosomes are shown in order of size from left to right and top to bottom. An interactive version of this figure may be viewed at https://genome-note-higlass.tol.sanger.ac.uk/l/?d=W8YFQjcZTti-GilSXDwOow.

Manual assembly curation corrected 235 missing joins or mis-joins and 214 haplotypic duplications, reducing the assembly length by 3.15% and the scaffold number by 41.69%, and decreasing the scaffold N50 by 2.16%. The final assembly has a total length of 1,272.30 Mb in 371 sequence scaffolds with a scaffold N50 of 411.9 Mb ( Table 2). The total count of gaps in the scaffolds is 960. The snail plot in Figure 2 provides a summary of the assembly statistics, while the distribution of assembly scaffolds on GC proportion and coverage is shown in Figure 3. The cumulative assembly plot in Figure 4 shows curves for subsets of scaffolds assigned to different phyla. Most (99.15%) of the assembly sequence was assigned to 4 chromosomal-level scaffolds. Chromosome-scale scaffolds confirmed by the Hi-C data are named in order of size ( Figure 5; Table 3). The order and orientation of contigs is uncertain along Chromosome 1 between 252 Mb and 260 Mb. While not fully phased, the assembly deposited is of one haplotype. Contigs corresponding to the second haplotype have also been deposited. The mitochondrial genome was also assembled and can be found as a contig within the multifasta file of the genome submission.

**Table 2.** Genome assembly data for *Tipula vernalis*, idTipVern1.1.

Genome assembly		
Assembly name	idTipVern1.1	
Assembly accession	GCA_958295665.1	
*Accession of alternate haplotype*	*GCA_958295445.1*	
Span (Mb)	1,272.30	
Number of contigs	1,332	
Contig N50 length (Mb)	3.3	
Number of scaffolds	371	
Scaffold N50 length (Mb)	411.9	
Longest scaffold (Mb)	463.88	
Assembly metrics *		*Benchmark*
Consensus quality (QV)	58.0	*≥ 50*
*k*-mer completeness	99.99%	*≥ 95%*
BUSCO **	C:94.5%[S:92.8%,D:1.6%], F:1.0%,M:4.5%,n:3,285	*C ≥ 95%*
Percentage of assembly mapped to chromosomes	99.15%	*≥ 95%*
Sex chromosomes	Not identified	*localised homologous pairs*
Organelles	Mitochondrial genome: 16.01 kb	*complete single alleles*
Genome annotation of assembly GCA_958295665.1 at Ensembl		
Number of protein-coding genes	13,911	
Number of non-coding genes	3,133	
Number of gene transcripts	23,847	

* Assembly metric benchmarks are adapted from column VGP-2020 of “Table 1: Proposed standards and metrics for defining genome assembly quality” from Rhie *et al.* (2021). ** BUSCO scores based on the diptera_odb10 BUSCO set using version 5.3.2. C = complete [S = single copy, D = duplicated], F = fragmented, M = missing, n = number of orthologues in comparison. A full set of BUSCO scores is available at https://blobtoolkit.genomehubs.org/view/idTipVern1_1/dataset/idTipVern1_1/busco.

**Table 3.** Chromosomal pseudomolecules in the genome assembly of *Tipula vernalis*, idTipVern1.

INSDC accession	Name	Length (Mb)	GC%
OY282558.1	1	463.88	33
OY282559.1	2	411.92	33
OY282560.1	3	371.1	33
OY282561.1	4	14.61	33
OY282562.1	MT	0.02	23

The estimated Quality Value (QV) of the final assembly is 58.0 with *k*-mer completeness of 99.99%, and the assembly has a BUSCO v5.3.2 completeness of 94.5% (single = 92.8%, duplicated = 1.6%), using the diptera_odb10 reference set ( *n* = 3,285).

Metadata for specimens, BOLD barcode results, spectra estimates, sequencing runs, contaminants and pre-curation assembly statistics are given at https://links.tol.sanger.ac.uk/species/2741129.

## Genome annotation report

The *Tipula vernalis* genome assembly (GCA_958295665.1) was annotated at the European Bioinformatics Institute (EBI) on Ensembl Rapid Release. The resulting annotation includes 23,847 transcribed mRNAs from 13,911 protein-coding and 3,133 non-coding genes ( Table 2; https://rapid.ensembl.org/Tipula_vernalis_GCA_958295665.1/Info/Index). The average transcript length is 17,465.22. There are 1.40 coding transcripts per gene and 3.95 exons per transcript.

## Methods

### Sample acquisition

Adult specimens of *Tipula vernalis* were collected from Wigmore Park, Luton, England, UK (latitude 51.88, longitude –0.37) on 2020-05-05. The specimen was netted and then kept in the fridge for 2 h before posting (to be delivered on 2020-05-07). The specimen was collected by Olga Sivell (Natural History Museum, London) and identified by Duncan Sivell (Natural History Museum, London) and preserved on dry ice. One specimen was used for genome sequencing (specimen ID NHMUK014111012, ToLID idTipVern1) and another specimen was used for Hi-C sequencing (specimen ID NHMUK 014111014, ToLID idTipVern2).

### Nucleic acid extraction

The workflow for high molecular weight (HMW) DNA extraction at the Wellcome Sanger Institute (WSI) Tree of Life Core Laboratory includes a sequence of core procedures: sample preparation and homogenisation, DNA extraction, fragmentation and purification. Detailed protocols are available on protocols.io ( Denton *et al.*, 2023b). In sample preparation, the idTipVern1 sample was weighed and dissected on dry ice ( Jay *et al.*, 2023) and tissue from the head and thorax was homogenised using a PowerMasher II tissue disruptor ( Denton *et al.*, 2023a).

RNA was extracted from abdomen tissue of idTipVern1 in the Tree of Life Laboratory at the WSI using the RNA Extraction: Automated MagMax™ *mir*Vana protocol ( do Amaral *et al.*, 2023). The RNA concentration was assessed using a Nanodrop spectrophotometer and a Qubit Fluorometer using the Qubit RNA Broad-Range Assay kit. Analysis of the integrity of the RNA was done using the Agilent RNA 6000 Pico Kit and Eukaryotic Total RNA assay.

### Hi-C preparation

Head and thorax tissue of the idTipVern2 sample was processed at the WSI Scientific Operations core, using the Arima-HiC v2 kit. In brief, frozen tissue (stored at –80 °C) was fixed, and the DNA crosslinked using a TC buffer with 22% formaldehyde. After crosslinking, the tissue was homogenised using the Diagnocine Power Masher-II and BioMasher-II tubes and pestles. Following the kit manufacturer's instructions, crosslinked DNA was digested using a restriction enzyme master mix. The 5’-overhangs were then filled in and labelled with biotinylated nucleotides and proximally ligated. An overnight incubation was carried out for enzymes to digest remaining proteins and for crosslinks to reverse. A clean up was performed with SPRIselect beads prior to library preparation.

### Library preparation and sequencing

Library preparation and sequencing were performed at the WSI Scientific Operations core. Pacific Biosciences HiFi circular consensus DNA sequencing libraries were prepared using the PacBio Express Template Preparation Kit v2.0 (Pacific Biosciences, California, USA) as per the manufacturer's instructions. The kit includes the reagents required for removal of single-strand overhangs, DNA damage repair, end repair/A-tailing, adapter ligation, and nuclease treatment. Library preparation also included a library purification step using AMPure PB beads (Pacific Biosciences, California, USA) and size selection step to remove templates <3kb using AMPure PB modified SPRI. DNA concentration was quantified using the Qubit Fluorometer v2.0 and Qubit HS Assay Kit and the final library fragment size analysis was carried out using the Agilent Femto Pulse Automated Pulsed Field CE Instrument and gDNA 165kb gDNA and 55kb BAC analysis kit. Samples were sequenced using the Sequel IIe system (Pacific Biosciences, California, USA). The concentration of the library loaded onto the Sequel IIe was between 40–135 pM. The SMRT link software, a PacBio web-based end-to-end workflow manager, was used to set-up and monitor the run, as well as perform primary and secondary analysis of the data upon completion.

Poly(A) RNA-Seq libraries were constructed using the NEB Ultra II RNA Library Prep kit, following the manufacturer’s instructions. RNA sequencing was performed on the Illumina NovaSeq 6000 instrument.

For Hi-C library preparation, DNA was fragmented to a size of 400 to 600 bp using a Covaris E220 sonicator. The DNA was then enriched, barcoded, and amplified using the NEBNext Ultra II DNA Library Prep Kit following manufacturers’ instructions. The Hi-C sequencing was performed using paired-end sequencing with a read length of 150 bp on an Illumina NovaSeq 6000 instrument.

### Genome assembly, curation and evaluation

***Assembly***

The HiFi reads were first assembled using Hifiasm ( Cheng *et al.*, 2021) with the --primary option. Haplotypic duplications were identified and removed using purge_dups ( Guan *et al.*, 2020). The Hi-C reads were mapped to the primary contigs using bwa-mem2 ( Vasimuddin *et al.*, 2019). The contigs were further scaffolded using the provided Hi-C data ( Rao *et al.*, 2014) in YaHS ( Zhou *et al.*, 2023) using the --break option. The scaffolded assemblies were evaluated using Gfastats ( Formenti *et al.*, 2022), BUSCO ( Manni *et al.*, 2021) and MERQURY.FK ( Rhie *et al.*, 2020).

The mitochondrial genome was assembled using MitoHiFi ( Uliano-Silva *et al.*, 2023), which runs MitoFinder ( Allio *et al.*, 2020) and uses these annotations to select the final mitochondrial contig and to ensure the general quality of the sequence.

***Assembly curation***

The assembly was decontaminated using the Assembly Screen for Cobionts and Contaminants (ASCC) pipeline (article in preparation). Manual curation was primarily conducted using PretextView ( Harry, 2022), with additional insights provided by JBrowse2 ( Diesh *et al.*, 2023) and HiGlass ( Kerpedjiev *et al.*, 2018). Scaffolds were visually inspected and corrected as described by Howe *et al.* (2021). Any identified contamination, missed joins, and mis-joins were corrected, and duplicate sequences were tagged and removed. The entire process is documented at https://gitlab.com/wtsi-grit/rapid-curation (article in preparation).

***Evaluation of the final assembly***

A Hi-C map for the final assembly was produced using bwa-mem2 ( Vasimuddin *et al.*, 2019) in the Cooler file format ( Abdennur & Mirny, 2020). To assess the assembly metrics, the *k*-mer completeness and QV consensus quality values were calculated in Merqury ( Rhie *et al.*, 2020). This work was done using the “sanger-tol/readmapping” ( Surana *et al.*, 2023a) and “sanger-tol/genomenote” ( Surana *et al.*, 2023b) pipelines. The genome readmapping pipelines were developed using the nf-core tooling ( Ewels *et al.*, 2020), use MultiQC ( Ewels *et al.*, 2016), and make extensive use of the Conda package manager, the Bioconda initiative ( Grüning *et al.*, 2018), the Biocontainers infrastructure ( da Veiga Leprevost *et al.*, 2017), and the Docker ( Merkel, 2014) and Singularity ( Kurtzer *et al.*, 2017) containerisation solutions. The genome was also analysed within the BlobToolKit environment ( Challis *et al.*, 2020) and BUSCO scores ( Manni *et al.*, 2021) were calculated.

Table 4 contains a list of relevant software tool versions and sources.

**Table 4.** Software tools: versions and sources.

Software tool	Version	Source
BlobToolKit	4.2.1	https://github.com/blobtoolkit/blobtoolkit
BUSCO	5.3.2	https://gitlab.com/ezlab/busco
bwa-mem2	2.2.1	https://github.com/bwa-mem2/bwa-mem2
Cooler	0.8.11	https://github.com/open2c/cooler
Gfastats	1.3.6	https://github.com/vgl-hub/gfastats
Hifiasm	0.16.1-r375	https://github.com/chhylp123/hifiasm
HiGlass	1.11.6	https://github.com/higlass/higlass
Merqury.FK	d00d98157618f4e8d1a9 190026b19b471055b22e	https://github.com/thegenemyers/MERQURY.FK
MitoHiFi	2	https://github.com/marcelauliano/MitoHiFi
PretextView	0.2	https://github.com/wtsi-hpag/PretextView
purge_dups	1.2.3	https://github.com/dfguan/purge_dups
sanger-tol/ genomenote	v1.0	https://github.com/sanger-tol/genomenote
sanger-tol/ readmapping	1.1.0	https://github.com/sanger-tol/readmapping/ tree/1.1.0
Singularity	3.9.0	https://github.com/sylabs/singularity
YaHS	1.1a.2	https://github.com/c-zhou/yahs

### Genome annotation

The Ensembl Genebuild annotation system ( Aken *et al.*, 2016) was used to generate annotation for the *Tipula vernalis* assembly (GCA_958295665.1) in Ensembl Rapid Release at the EBI. Annotation was created primarily through alignment of transcriptomic data to the genome, with gap filling via protein-to-genome alignments of a select set of proteins from UniProt ( UniProt Consortium, 2019).

### Wellcome Sanger Institute – Legal and Governance

The materials that have contributed to this genome note have been supplied by a Darwin Tree of Life Partner. The submission of materials by a Darwin Tree of Life Partner is subject to the **‘Darwin Tree of Life Project Sampling Code of Practice’**, which can be found in full on the Darwin Tree of Life website here. By agreeing with and signing up to the Sampling Code of Practice, the Darwin Tree of Life Partner agrees they will meet the legal and ethical requirements and standards set out within this document in respect of all samples acquired for, and supplied to, the Darwin Tree of Life Project.

Further, the Wellcome Sanger Institute employs a process whereby due diligence is carried out proportionate to the nature of the materials themselves, and the circumstances under which they have been/are to be collected and provided for use. The purpose of this is to address and mitigate any potential legal and/or ethical implications of receipt and use of the materials as part of the research project, and to ensure that in doing so we align with best practice wherever possible. The overarching areas of consideration are:

•   Ethical review of provenance and sourcing of the material

•   Legality of collection, transfer and use (national and international)

Each transfer of samples is further undertaken according to a Research Collaboration Agreement or Material Transfer Agreement entered into by the Darwin Tree of Life Partner, Genome Research Limited (operating as the Wellcome Sanger Institute), and in some circumstances other Darwin Tree of Life collaborators.

## Data Availability

European Nucleotide Archive: *Tipula vernalis.* Accession number PRJEB57430; https://identifiers.org/ena.embl/PRJEB57430 ( Wellcome Sanger Institute, 2023). The genome sequence is released openly for reuse. The *Tipula vernalis* genome sequencing initiative is part of the Darwin Tree of Life (DToL) project. All raw sequence data and the assembly have been deposited in INSDC databases. Raw data and assembly accession identifiers are reported in Table 1 and Table 2.

## Author information

Members of the Wellcome Sanger Institute Tree of Life Management, Samples and Laboratory team are listed here: https://doi.org/10.5281/zenodo.12162482.

Members of Wellcome Sanger Institute Scientific Operations: Sequencing Operations are listed here: https://doi.org/10.5281/zenodo.12165051.

Members of the Wellcome Sanger Institute Tree of Life Core Informatics team are listed here: https://doi.org/10.5281/zenodo.12160324.

Members of the Tree of Life Core Informatics collective are listed here: https://doi.org/10.5281/zenodo.12205391.

Members of the Darwin Tree of Life Consortium are listed here: https://doi.org/10.5281/zenodo.4783558.
