# Supplementary material for: The genome sequence of a cranefly Tipula ( Lunatipula) vernalis Meigen, 1804
Source: Wellcome Open Res. 2024 Nov 7;9:658. [Version 1] doi: 10.12688/wellcomeopenres.23203.1 (PMC11803394; doi:10.12688/wellcomeopenres.23203.1)
Supplement: Peer review history

# Reviewer report: Reviewer response for version 1

**DOI:** 10.21956/wellcomeopenres.25553.r113765

**Published:** 2024-12-16

**Authors:** Komal Kumar Bollepogu Raja

**Affiliations:** 1 Baylor College of Medicine, Houston, TX, USA

The article by Sivell et al. presents a detailed genome assembly of *Tipula vernalis* *. *The authors employed advanced sequencing technologies for the assembly and the methodology is technically sound and comprehensive, with clear descriptions of the bioinformatic tools used for genome assembly. This allows for reproducibility. The quality assessments, such as coverage, QV and the 94.5% BUSCO score are impressive, robust and considered standard for genome assembly. The percent of assembly mapped to chromosomes is very high and commendable. Finally, the number of genes and transcripts are also presented. Overall, the data in this manuscript is high quality and is a useful resource for experts as well as for other researchers. I approve indexing of these data.

Are sufficient details of methods and materials provided to allow replication by others?

Yes

Is the rationale for creating the dataset(s) clearly described?

Yes

Are the datasets clearly presented in a useable and accessible format?

Yes

Are the protocols appropriate and is the work technically sound?

Yes

Reviewer Expertise:

Single cell omics, genomics, bioinformatics and Neurobiology

I confirm that I have read this submission and believe that I have an appropriate level of expertise to confirm that it is of an acceptable scientific standard.

# Reviewer report: Reviewer response for version 1

**DOI:** 10.21956/wellcomeopenres.25553.r114745

**Published:** 2024-12-10

**Authors:** Michael Ritchie

**Affiliations:** 1 University of St Andrews, St Andrews, Scotland, UK

This reports the genome of a cranefly. The methods are all appropriate and reported well. The results are notably clean and well presented and have been carried out to a high standard. The data will be of interest to all entomologists and dipterists and are well worth indexing. The only comment perhaps worth a minor revision is the finding of 4 large pseudochromosomes containing >99% of the sequence, and a lot of small contigs. What is expected? A quick search revealed that most UK craneflies have 6 autosomes plus an XY sex pair, so this is unexpected? Is there more recent karyoptypic work? This could be elaborated in a few sentences.

 Henderson, S. A. and T. Parsons (1963). "CHROMOSOMES OF 11 SPECIES OF TIPULID." Caryologia 16(2): 337

Are sufficient details of methods and materials provided to allow replication by others?

Yes

Is the rationale for creating the dataset(s) clearly described?

Yes

Are the datasets clearly presented in a useable and accessible format?

Yes

Are the protocols appropriate and is the work technically sound?

Yes

Reviewer Expertise:

Evolutionary genetics

I confirm that I have read this submission and believe that I have an appropriate level of expertise to confirm that it is of an acceptable scientific standard.

## References

[1] : CHROMOSOMES OF 11 SPECIES OF TIPULID. *Caryologia 16(2): 337* .1963;
